# Study on medicinal food plants in the Gaoligongshan Biosphere Reserve, the richest biocultural diversity center in China

**DOI:** 10.1186/s13002-023-00638-9

**Published:** 2024-01-15

**Authors:** Zhuo Cheng, Shuyan Lin, Ziyi Wu, Chen Lin, Qing Zhang, Congli Xu, Jiahua Li, Chunlin Long

**Affiliations:** 1 Key Laboratory of Ecology and Environment in Minority Areas (Minzu University of China), National Ethnic Affairs Commission of China, Beijing, 100081 China; 2 College of Life and Environmental Sciences, Minzu University of China, Beijing, 100081 China; 3 Institute of National Security Studies, Minzu University of China, Beijing, 100081 China; 4 Yunnan Gaoligongshan National Nature Reserve (Baoshan Bureau), Yunnan, 678000 China; 5 Yunnan Gaoligongshan National Nature Reserve (Longyang Branch of Baoshan Bureau), Yunnan, 678000 China

**Keywords:** Medicinal food plants, The Gaoligongshan area, Ethnobotany, Traditional knowledge

## Abstract

**Background:**

Traditional knowledge associated with medicinal food plants (MFPs) plays a vital role in fighting hidden hunger and safeguarding the health of local people. MFPs resources are abundant in the Gaoligongshan area, a biosphere reserve with the richest biocultural diversity in China. Local people of different linguistic groups also have rich traditional botanical knowledge. However, there are still few comprehensive and systematic studies on MFPs there.

**Methods:**

Ethnobotanical investigation including market survey, semi-structured interviews, free listing and key informant interviews was conducted in the Gaoligongshan area, Western Yunnan, Southwest China. A total of 13 local farmers’ markets were selected and information about medicinal food plants, including food categories, medicinal and edible parts, modes of consumption, medicinal effects, and distribution were collected. The relative occurrence frequency (RFO) and cultural food significance index (CFSI) were calculated to identify the culturally significant MFPs.

**Results:**

A total of 184 species of MFPs, belonging to 83 families, were collected in the Gaoligongshan area, including vegetables (77), medicinal diets (26), fruits (25), spices (18), herbal tea (13), tea substitutes (11), substitutes for staple food (8), nuts (5), oils and fats (4), and dye material (1). The most frequently used families were Fabaceae, Asteraceae and Apiaceae, with 11, 10, and 9 species, respectively. The most frequently used plant parts were the stems, followed by fruits and leaves. Based on the evaluation results of the CFSI and RFO indices, 18 species of MFPs with magnificent local cultural importance have been screened out, such as *Houttuynia cordata*, *Eryngium foetidum*, *Sechium edule*, *Centella asiatica* and *Pseudocydonia sinensis*.

**Conclusion:**

These findings have guiding significance for conservation of traditional knowledge associated with MFPs and facilitation of scientific utilization of MFPs to meet local people’s needs for a healthy life.

**Supplementary Information:**

The online version contains supplementary material available at 10.1186/s13002-023-00638-9.

## Background

The greatest global challenge is to ensure that all people have access to nutritious food and healthy medicine. Medicinal food plants, or medicinal dietary plants, refer to plants that can be eaten and also be used as medicine to prevent and cure diseases [1–4]. For remote areas in developing countries, traditional knowledge of medicinal food plants plays a vital role in fighting hidden hunger and safeguarding the health of local people [5, 6].

At present, conducting ethnobotanical surveys of MFPs resources has attracted the interest of many ethnic botanists and has become the focus of research [7–9]. Especially in edible plants, international ethnobotanical research on edible plants is mainly concentrated in Europe, Africa and Asia, such as Italy, Poland, East Africa, the southern Sahara and the southern foothills of the Himalayas, while relatively little research in North America, South America and Oceania [10]. There are many domestic studies on MFPs, mainly concentrated on the utilization of plants by ethnic minorities in minority areas, such as Naxi, Hani, Mongolian, Tibetan and Yi. [11–16]. These studies document the traditional knowledge of edible plants and use different quantitative methods to assess the local importance of wild food plants, which play an essential role in protecting traditional knowledge and the sustainable use of WEPs and finding the most widely consumed varieties and analyzing their nutritional value [17, 18]. The nutritional analysis results will provide clues for finding excellent germplasm resources, help ensure the diversity of diet and achieve food security [19, 20].

Market research is a commonly used method in ethnobotanical research associated with MFPs [21]. Open-air markets are great places to gain a deep understanding of MFPs: A great variety of MFPs are traded in local markets. Besides, the demand of local people and their unique traditional culture can be easily reflected by the number of available MFPs [22, 23]. Even though there are tons of advanced grocery and supermarkets in modernized societies, open-air markets still remain important place of trading plant for both urban and rural dwellers. In recent years, some ethnobotanists have conducted a deep study on the Dragon Boat Festival in southwest China. These studies have used market survey methods to investigate the Dragon Boat Festival in Jianghua County of Hunan, Gongcheng County, Guangxi, and Qianxinan County of Guizhou and found that the local Yao and Zhuang ethnic groups sell hundreds of herbs in the medicine market during the Dragon Boat Festival [24–27]. Apart from domestic research, there is also an abundance of research using market survey abroad. Taking South America as an example, various market surveys have been done on Bolivia, Peru and Brazil markets [28–31]. Since the healers as well as the laypeople purchase their medicinal plants in local markets, researchers found local markets valuable places for having a deep insight into their specific medicinal plants which contain rich medical knowledge passed down from their ancestors in each different ethnic group. Market research is also a significant approach in Europe and Asian. Researchers adopted this method to figure out association between market trend and data results in Greece, and other researchers took this measure to fill vacancies in products sold in the markets in Poland [32, 33]. Additionally, some researchers used market research to access the diverse wild vegetable resources sold at the local markets of Manipur throughout different seasons in India [34], researchers used this method to draw a conclusion about medicinal plants on the markets composition which mainly was imported from outside in Iraq [35]. Researchers in Pakistan observed local medicine plants status quo and came up with some causing reasons like increased marketing pressure on medicinal plants, lack of job opportunities in the area, non-sustainable harvesting methods like digging of whole plant and increased population of the area in this way as well [36]. Besides, an ethnobotanical survey on the medicinal plant species marketed in Iran was conducted in order to document traditional medicinal knowledge and application of medicinal plants [37].

China is a country with a long history, rich biodiversity and diverse ethnic cultures [38]. Over the long history and development of different linguistic groups, they have accumulated traditional knowledge of using MFPs to treat diseases and to resist the harsh natural environment [39].

The Gaoligong Mountains or the Gaoligongshan area (24°34’-28°22’N, 97°30’-99°30’E) refers to the diverging mountains between the Nujiang River (upstream of Salween River) and Dulong River (a branch of Irrawaddy River) and the areas on both sides of the mountains. The Gaoligong Mountains have been designated as a biosphere reserve by United Nations Educational, Scientific and Cultural Organization since 2010. It is also the core area of the Three Rivers Parallel World Natural Heritage site and has been listed as one of the world’s 25 new biodiversity hotspots [40, 41]. The Gaoligongshan area not only boasts the highest number of species per unit area in China, but also is one of the areas with the richest cultural diversity in China. More than ten ethnic groups live in and enjoy a rich culture of medicinal food plants [42].

Previous ethnobotanical studies in the Gaoligong Mountains have proven that local people have rich traditional knowledge about medicinal food plants [40, 43–47]. In addition, some studies on *Maianthemum atropurpurea*, an important medicinal food plant in the Gaoligong Mountains, show that it is a functional wild vegetable that can meet the requirements of modern healthy diet [48]. Another study on lacquer oil from the drupes of *Toxicodendron vernicifluum* preliminarily verified the rationality and scientificity of lacquer oil in treating gynaecopathia ailment [49]. However, systematical and comprehensive studies on medicinal food plants of the Gaoligong Mountains are very limited.

With the acceleration of population aging and the increase of people with sub-health and chronic diseases, the codified knowledge from traditional Chinese medicine therapy has been received considerable attention [50]. However, with the infiltration of mainstream cultures, the destruction of the natural environment and the expansion of urbanization, the traditional knowledge associated with MFPs is facing the danger of assimilation and loss [51–54]. Many studies from various regions have found that sociocultural factors are the main drivers of reduced consumption of medicinal and edible plants [55, 56]. Other studies pointed out that the main drivers of decreased abundance are perceived to be land-use change and direct exploitation of medicinal and edible plants. These changes have potential negative implications on food systems from local to global scales [57]. The resources of MFPs are constantly threatened by various natural factors and human activities. Furthermore, global climate change leads to various extreme weather events, for example thunderstorms, mudslides and flash floods, which significantly contribute to largescale plant deaths. In addition, various human activities (single-crop cultivation, habitat destruction, excessive harvesting, overgrazing, etc.) also pose a considerable threat to wild plant resources.

A comprehensive study should be carried out to document the traditional knowledge of MFPs. Besides, the endangered traditional knowledge should be identified and evaluated, which will help promote regional economic development and ensure the conservation and sustainable use of MFPs. The objectives of this study were to: (1) conduct a comprehensive study on MFPs used by local people living in the Gaoligongshan area, (2) record the traditional knowledge associated with MFPs, (3) identify species of important cultural significance to local people, and (4) analyze the opportunities and challenges for the protection of MFPs.

## Methods

### Study site

The Gaoligong Mountains (24°40′ to 28°30′ N, 97°30′ to 99°30′ E) is located at the junction of southwestern Yunnan and northern Myanmar. The administrative region covers Baoshan (Tengchong, Longyang and Longling), Nujiang (Lushui, Fugong and Gongshan) and Linzhi (Chayu) of China, and Kachin State of northern Myanmar [41, 45]. Due to the special geological history and unique ecological environment, there are diverse biological species in the Gaoligong Mountain. It has been recognized as the richest area in biodiversity of China and listed as one of the world’s 25 biodiversity hotspots [41]. Up to now, 5139 species of seed plants have been recorded in the Gaoligong Mountains. The richness of endemic species is higher than most areas in the country. The Gaoligongshan region is also very rich in medicinal plant resources, with as many as 1298 species, including about 300 species traditionally used by ethnic groups [58].

The Gaoligongshan area is also one of the richest centers of cultural diversity in China. Nearly 2/3 of the ethnic minorities in Yunnan Province are concentrated in this area. The Bai, Naxi, Lisu, Tibetan, Nu, Dulong, Pumi, Wa, Achang and other ethnic minorities together with Han Chinese have lived in the area and showed diverse traditional customs and cultures [40]. The economic and social status of the Gaoligong Mountains is in developing stage, with low productivity. Although it is one of the poorest areas in Yunnan, the local people of various linguistic groups have very rich traditional knowledge associated with biodiversity. Such a variety of life forms and national traditional cultures in the Gaoligong Mountains have created its world-renowned biocultural diversity.

**Fig. 1:**
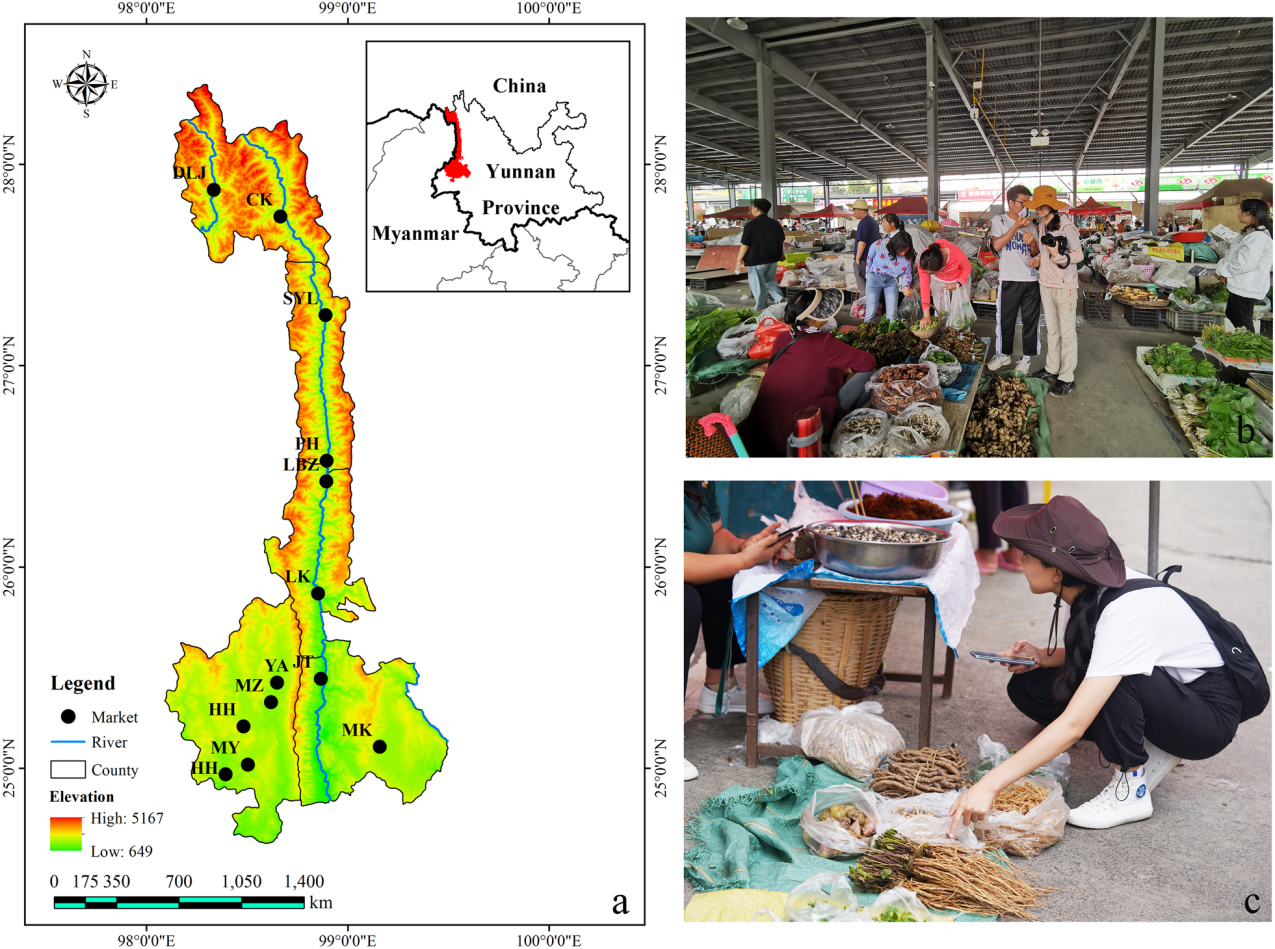
Study site. **a** Location of study sites; **b** Jietou Famers’ market; **c** Semi-structured interviews

The study area included five county-level administrative units, including three in Nujiang Lisu Autonomous Prefecture (Gongshan Dulong and Nu Autonomous County, Fugong County, and Lushui City), and two in Baoshan City (Longyang District, and Tengchong City) (Fig. 1). Based on the natural and cultural conditions of every conties in the Gaoligongshan area, 13 representative local farmers' markets belong to different ethnic groups were uniformly selected in the area, as shown in Table 1.

**Table 1:** The location and linguistic groups of markets investigated

Name	Abbreviation	Latitude	Longitude	Subordinate county or city	Linguistic group
Dulongjiang farmer's market	DLJ	27°52′23.32"	98°20′8.27"	Gongshan County	Dulong mainly
Gongshan farmer's market	GS	27°44′27.02"	98°39′58.6"	Gongshan County	Multiple groups
Luobenzhuo Bai Township market	LBZ	26°25′26.48"	98°53′41.95"	Fugong County	Bai mainly
Shiyueliang farmer's market	SYL	27°14′59.88"	98°53′26.53"	Fugong County	Multiple groups
Pihe Nu market	PH	26°31′38.51"	98°53′48.98"	Fugong County	Nu mainly
Liuku farmer's market	LK	25°52′10.67"	98°51′13.4"	Lushui City	Lisu mainly
Nanyuan farmer's market	NY	25°6′23.53"	99°9′39.17"	Longyang District	Multiple groups
Mangkuan farmer's market	MK	25°26′42.73"	98°52′6.44"	Longyang District	Multiple groups
Jietou farmer's market	JT	25°25′33.55"	98°39′0.96"	Tengchong City	Multiple groups
Yongan farmer's market	YA	25°19′42.06"	98°37′13.61"	Tengchong City	Multiple groups
Mazhan farmer's market	MZ	25°12′30.72"	98°28′59.84"	Tengchong City	Multiple groups
Hehua farmer's market	HH	24°58′10.55"	98°23′40.9"	Tengchong City	Multiple groups
Manyi farmer's market	MY	25°1′5.51"	98°30′17.53"	Tengchong City	Multiple groups

### Literature research

Through investigating local chronicles and flora, the general information was collected, including topography, climatic conditions, and vegetation of the Gaoligong Mountains, history, customs, religious beliefs, and social culture of the local people. Such information helped to choose suitable markets and seasons for field surveys before the ethnobotanical investigations were implemented.

### Market survey

Market surveys included semi-structured interviews, key informant interviews and participatory observation. During the market investigation, some stallholder was invited to list all medicinal dietary plants that are still regularly used. The interviews consisted of two parts, the first part was about the basic situation of the informants (linguistic group, age, education, occupation), and the other part contained questions related to recording detailed information on MFPs, including their local names, availability, used part, processing methods, frequency of use, mouthfeel, whether they are used as a medical diet, application methods, medicinal effects, and other uses.

### Voucher specimen collection and identification

The voucher specimen of each medicinal food plant was collected, and deposited in the herbarium at Minzu University of China. For the identification of plants, the voucher specimens were studied and compared with reference books (*Flora Republicae Popularis Sinicae* and *Flora of China*) and electronic online resources (http://www.iplant.cn/ and https://powo.science.kew.org).

### Quantitative ethnobotanical research

An improvement is made on the basis of the relative frequency of citation (RFC), using relative occurrence frequency (RFO) index to quantify the frequency of certain MFPs in the market. N represents the number of all markets investigated. RFO ranges from 0 to 1.

$${\text{RFO}} = \frac{{{\text{FO}}}}{{\text{N}}}$$

It is calculated by dividing the number of markets in which the medicinal diet plant appears by the total number of markets. FO refers to the number of markets in which a particular herbal diet plant appears. N represents the number of all the markets investigated. RFO value ranges from 0 to 1. The higher the RFO value, the more widespread the use of the plant in the region. The prevalence of each medicinal diet plant is expressed by the value of the RFO, which allows all medicinal diet plants mentioned in the survey to be listed in the order of their prevalence sizes.

The cultural food significance index (CFSI) is calculated to evaluate the cultural significance of MFPs [59].

$${\text{CFSI}} = {\text{RFO}} \times {\text{AI}} \times {\text{FUI}} \times {\text{PUI}} \times {\text{MFFI}} \times {\text{TSAI}} \times {\text{FMRI}} \times {1}0^{{ - {2}}}$$

It is composed of seven factors including the relative occurrence frequency (RFO) index, availability index (AI), frequency of utilization index (FUI), parts used index (PUI), multifunctional food use index (MFFI), taste score evaluation index (TSAI) and food-medicinal role index (FMRI). The larger the CFSI is, the more important the role of this plant is in diet.

## Results

### Diversity of medicinal food plants

The survey results showed that there were 184 species of medicinal food plants in Gaoligong Mountains area, including 173 angiosperm species, 1 gymnosperm species, 8 fern species, and 2 lichen species. Among the 13 markets surveyed, the Mangkuan Market in Longyang District, Baoshan City has the most medicinal food plants with 80 species, and the Luobenzhuo Market in Fugong County has the least, only 34 species (Table 2). Other information of all MFPs is provided in the supplementary materials (Additional file 1: Table S1).

**Table 2:** The inventory of medicinal food plants in Gaoligong Mountains area

Scientific name	Chinese name	Family	Food categories	Part used and mode of consumption	Medicinal part and processing method	Medicinal effect	RFO	CFSI	IUCN	Voucher number
**Angiosperm**										
*Asystasia neesiana* (Wall.) Nees	白接骨	Acanthaceae	Vegetables	Tender stem, tender leaf; boiled or stir-fried	Whole plant; decoction	Hemostasis	0.15	3.24		DLZ0086
*Saurauia napaulensis* DC	尼泊尔水东哥	Actinidiaceae	Fruits	Fruit; eaten freshly	Root, fruit; decoction	Dispersing blood stasis, swelling, fracture, fall injury	0.38	1.35	LC	DLZ0046, DXB0013
*Sagittaria trifolia* subsp. *leucopetala* (Miquel) Q. F. Wang	华夏慈姑	Alismataceae	Vegetables	Bulb; stir-fried	Bulb; decoction, external application	Activating blood, cooling blood, relieving cough	0.62	14.04		
*Spinacia oleracea* L	菠菜	Amaranthaceae	Vegetables	Whole plant; boiled or stir-fried	Whole plant; decoction	Blurred vision, anemia, constipation	0.31	32.40		
*Amaranthus tricolor* L	苋	Amaranthaceae	Vegetables	Tender stem, tender leaf; stir-fried	Whole plant; decoction	Clearing heat and detoxifying, bright eyes	0.46	9.36		
*Chenopodium album* L	藜	Amaranthaceae	Vegetables	Tender stem, tender leaf; boiled or stir-fried	Whole plant; decoction	Dysentery, diarrhea, skin itching	0.08	1.20		DXB0102
*Amaranthus cruentus* L	老鸦谷	Amaranthaceae	Vegetables	Tender stem, tender leaf; stir-fried	Whole plant; decoction	Clearing heat and detoxifying, dysentery, jaundice	0.23	1.17		
*Amaranthus retroflexus* L	反枝苋	Amaranthaceae	Vegetables	Tender stem, tender leaf; stir-fried	Whole plant; decoction	Diarrhea, dysentery, carbuncle swelling pain	0.08	0.78		GLG0050
*Allium hookeri* Thwaites	宽叶韭	Amaryllidaceae	Vegetables	Whole plant; boiled or stir-fried	Whole plant; decoction	Clearing heat and detoxifying, moistening lung and relieving cough	0.77	432.00		DXB0120
*Allium fistulosum* L	葱	Amaryllidaceae	Spices	Whole plant; boiled or stir-fried	Whole plant; decoction	Antisepsis and anti-inflammation	0.77	156.00		
*Allium sativum* L	蒜	Amaryllidaceae	Spices	Whole plant; boiled or stir-fried	Whole plant; decoction	Antisepsis and anti-inflammation	0.69	175.50		
*Allium cepa* L	洋葱	Amaryllidaceae	Vegetables	Bulb; stir-fried	Bulb; decoction	Antisepsis and anti-inflammation	0.46	47.39		
*Allium tuberosum* Rottler ex Sprengle	韭	Amaryllidaceae	Vegetables	Whole plant; boiled or stir-fried	Whole plant; decoction	Antisepsis and anti-inflammation	0.77	70.20		
*Allium prattii* C. H. Wright ex Hemsl	太白山葱	Amaryllidaceae	Vegetables	Whole plant; boiled or stir-fried	Whole plant; decoction	Antisepsis and anti-inflammation	0.15	1.35		
*Allium wallichii* Kunth	多星韭	Amaryllidaceae	Vegetables	Whole plant; boiled or stir-fried	Whole plant; decoction	Antisepsis and anti-inflammation	0.23	2.03		
*Toxicodendron vernicifluum* (Stokes) F. A. Barkl	漆	Anacardiaceae	Oils and fats	Fruit; press into oil	Seed; boiled with meat	Lactation, moon disease ( postpartum depression)	0.54	70.88	LC	DLZ0172, DXB0014
*Choerospondias axillaris* (Roxb.) B. L. Burtt & A. W. Hill	南酸枣	Anacardiaceae	Fruits	Fruit; eaten freshly	Fruit; decoction	Heart palpitations shortness of breath, restless mind	0.08	0.81	LC	GLG0051
*Eryngium foetidum* L	刺芹	Apiaceae	Spices	Aerial part; boiled or stir-fried	Whole plant; decoction	Diuresis, treatment of edema, snake bites	0.92	648.00		DXB0154
*Centella asiatica* (L.) Urban	积雪草	Apiaceae	Vegetables	Whole plant; boiled or stir-fried	Whole plant; decoction	Heat-clearing and detoxifying, jaundice, heat stroke, diarrhea, bruises	0.92	388.80	LC	GLG0052
*Foeniculum vulgare* Mill	茴香	Apiaceae	Spices	Whole plant; boiled or stir-fried, cold and dressed with sauce	Fruit; decoction	Balsam	0.62	630.00		GLG0062
*Ligusticum sinense* 'Chuanxiong'	川芎	Apiaceae	Medicinal diets	Rhizome; cook soup, fried with eggs	Root, rhizome; boiled with meat	Activating blood, relieving pain	0.62	192.00		GLG0054
*Oenanthe javanica* (Bl.) DC	水芹	Apiaceae	Vegetables	Tender stem, tender leaf; stir-fried, fried with eggs	Whole plant; decoction	Antihypertensive	0.38	81.00	LC	DLZ0062, DLZ0063
*Coriandrum sativum* L	芫荽	Apiaceae	Spices	Whole plant; stir-fried	Whole plant; decoction	Invigorating stomach, romoting digestion	0.15	54.00		GLG0055
*Pimpinella candolleana* Wight et Arn	杏叶防风	Apiaceae	Medicinal diets	Rhizome; cook soup	Root, rhizome; boiled with meat	Jaundice hepatitis, acute cholecystitis	0.23	13.50		GLG0058
*Hansenia weberbaueriana* (Fedde ex H. Wolff) Pimenov & Kljuykov	羌活	Apiaceae	Medicinal diets	Rhizome; cook soup	Stem, rhizome; boiled with meat	Wind cold cold cold, headache, rheumatism pain	0.08	1.24		GLG0061
*Angelica sinensis* (Oliv.) Diels	当归	Apiaceae	Medicinal diets	Rhizome; cook soup	Stem, rhizome; boiled with meat	Reinforcing blood and activating blood circulation, irregular menstruation, dysmenorrhea, drop injury	0.08	2.48		GLG0067
*Dregea volubilis* (L. f.) Benth. ex Hook. f	南山藤	Apocynaceae	Vegetables	Tender stem, tender leaf; stir-fried	Whole plant; decoction	Stomachache	0.08	0.90		GLG0031
*Colocasia esculenta* (L.) Schott	芋	Araceae	Staple food substitutes	Tuber; boiled or stir-fried, cook soup	Whole plant; decoction, external application	Mouth sores, burns, trauma bleeding	0.77	111.38	LC	DLZ0135
*Amorphophallus konjac* K. Koch	魔芋	Araceae	Staple food substitutes	Tuber; make gel-based foods	Stem; decoction, external application	Dispersing blood stasis to stop bleeding, detumescence and relieving pain, invigorating stomach and eliminating food	0.77	40.50		GLG0033
*Leucocasia gigantea* (Blume) Schott	大野芋	Araceae	Vegetables	Leaf stalk; boiled or stir-fried	Root, stem; decoction, external application	Falling injury, snakebite bite	0.15	4.50		GLG0035
*Eleutherococcus senticosus* (Ruprecht & Maximowicz) Maximowicz	刺五加	Araliaceae	Vegetables	Tender stem, tender leaf; stir-fried	Bark; decoction	Renal asthenia	0.23	13.50		GLG0039
*Aralia chinensis* L	黄毛楤木	Araliaceae	Vegetables	Tender stem, tender leaf; stir-fried	Bark, stem; decoction; external application	Gastritis, nephritis and rheumatic pain, knife wounds	0.62	10.80	VU	DLZ0166
*Trevesia palmata* (Roxb.) Vis	刺通草	Araliaceae	Vegetables	Tender stem, tender leaf; stir-fried	Leaf; decoction	Removing stasis and relieving pain, nourishing and strengthening, falling injury, trauma	0.08	0.90	LC	GLG0082
*Panax bipinnatifidus* Seemann	疙瘩七	Araliaceae	Medicinal diets	Rhizome; cook soup	Rhizome; boiled with meat	Activating blood circulation to remove blood stasis, falling injury	0.38	0.75		GLG0041
*Panax japonicus* (T. Nees) C. A. Meyer	竹节参	Araliaceae	Medicinal diets	Rhizome; cook soup	Rhizome; boiled with meat	Blood stasis hemostasis, detumescence pain, bruises, coughing	0.23	0.62		GLG0043
*Trachycarpus fortunei* (Hook.) H. Wendl	棕榈	Arecaceae	Vegetables	Inflorescence; stir-fried	Petiole, leaf sheath; decoction, external application	Convergent hemostasis, for hematuria, hematochezia	0.38	12.15		GLG0047
*Caryota obtusa* Griffith	董棕	Arecaceae	Staple food substitutes	Pith; processed into flour	Pith; decoction	Dysentery, indigestion, stomachache	0.15	0.34		DLZ0005
*Maianthemum atropurpureum* (Franchet) LaFrankie	高大鹿药	Asparagaceae	Vegetables	Tender stem, tender leaf; boiled or stir-fried	Stem, leaf; decoction	Clearing heat and detoxifying, lowering blood pressure	0.46	28.80		DXB0090
*Polygonatum sibiricum* Delar. ex Redoute	黄精	Asparagaceae	Medicinal diets	Rhizome; cook soup	Rhizome; decoction, external application, boiled with meat	Lung, kidney, spleen and stomach qi deficiency	0.23	3.38		DXB0101
*Hemerocallis citrina* Baroni	黄花菜	Asphodelaceae	Vegetables	Flower; boiled or stir-fried	Flower; dry	Invigorating stomach, diuresis, detumescence	0.08	3.24		
*Taraxacum mongolicum* Hand.-Mazz	蒲公英	Asteraceae	Vegetables	Whole plant; boiled or stir-fried	Whole plant; decoction	Heat-clearing and detoxifying, swelling and resolving masses, used for furuncle swelling, sore throat, jaundice	0.62	108.00		DLZ0016
*Arctium lappa* L	牛蒡	Asteraceae	Medicinal diets	Rhizome; cook soup	Root; boiled with meat	Colds, coughs, sore throats	0.23	12.15		GLG0048
*Sonchus oleraceus* L	苦苣菜	Asteraceae	Vegetables	Whole plant; boiled or stir-fried	Whole plant; decoction, external application	clearing away heat andtoxic materials	0.08	13.50		GLG0091
*Dichrocephala integrifolia* (Linnaeus f.) Kuntze	鱼眼草	Asteraceae	Vegetables	Tender stem,tender leaf; boiled or stir-fried	Whole plant; decoction	Anti-inflammatory, antidiarrheal, treatment of dyspepsia in children	0.15	12.48		GLG0099
*Crassocephalum crepidioides* (Benth.) S. Moore	野茼蒿	Asteraceae	Vegetables	Tender stem,tender leaf; boiled or stir-fried	Whole plant; decoction	Spleen, swelling, treatment of indigestion	0.23	16.20		GLG0092
*Cirsium japonicum* Fisch. ex DC	蓟	Asteraceae	Vegetables	Root; cook soup	Root; decoction	Cooling blood to stop bleeding, removing blood stasis and detoxification, used for hematuria, hematochezia, traumatic bleeding, carbuncle sore poison	0.31	13.50		GLG0093
*Tanacetum tatsienense* (Bureau & Franchet) K. Bremer & Humphries	川西小黄菊	Asteraceae	Medicinal diets	Whole plant; boiled with meat	Whole plant; boiled with meat	Activating blood, anti-inflammatory, analgesic, falling injury	0.15	1.20		GLG0015
*Pseudognaphalium affine* (D. Don) Anderberg	鼠曲草	Asteraceae	Vegetables	Tender stem, tender leaf; stir-fried	Stem, leaf; decoction	Antitussive, expectorant, treatment of asthma and bronchitis and non-infectious ulcers, trauma; oral administration to reduce blood pressure	0.08	4.32		GLG0016
*Chrysanthemum lavandulifolium* (Fischer ex Trautvetter) Makino	甘菊	Asteraceae	Tea substitute	Flower; dry, soaking in water	Flower; decoction	Clearing heat and detoxifying, colds	0.08	0.84		
*Helianthus tuberosus* L	菊芋	Asteraceae	Vegetables	Tuber; stir-fried	Root; decoction	Prevention of intestinal infection	0.08	1.17	LC	GLG0014
*Basella alba* L	落葵	Basellaceae	Vegetables	Tender stem, tender leaf; boiled or stir-fried	Whole plant; decoction	Facilitate excretion	0.08	5.40		
*Brassica oleracea* var. *gongylodes* Linnaeus	擘蓝	Brassicaceae	Vegetables	Bulb, tender leaf; cold and dressed with sauce,stir-fried, cook soup	Bulb; decoction	Digestion, duodenal ulcer	0.23	49.91		
*Brassica juncea* (Linnaeus) Czernajew	芥菜	Brassicaceae	Vegetables	Bulb; stir-fried	Leaf, seed; decoction	Digestion, duodenal ulcer	0.46	16.20		
*Capsella bursa-pastoris* (L.) Medic	荠	Brassicaceae	Vegetables	Whole plant; stir-fried	Whole plant; decoction	Diuresis, hemostasis, indigestion	0.08	4.68		
*Cardamine tangutorum* O. E. Schulz	紫花碎米荠	Brassicaceae	Vegetables	Tender stem, tender leaf; boiled or stir-fried	Whole plant; decoction	Tendon pain	0.38	21.60		GLG0097
*Brasenia schreberi* J. F. Gmel	莼菜	Cabombaceae	Vegetables	tender stem, tender leaf; stir-fried	Tender stem and leaf; decoction	Clearing away heat andtoxic materials	0.08	0.27	LC	
*Campanumoea javanica* Bl	金钱豹	Campanulaceae	Medicinal diets	Rhizome; cook soup	Rhizome; boiled with meat	Neurasthenia	0.08	0.84		
*Canna indica* 'Edulis'	蕉芋	Cannaceae	Vegetables	Tuber; boiled or stir-fried	Root; decoction	Diarrhea	0.38	40.50		GLG0022
*Valeriana jatamansi* Jones	蜘蛛香	Caprifoliaceae	Medicinal diets	Whole plant; cook soup	Whole plant; decoction, external application	Abdominal pain, diarrhea, dyspepsia, rheumatism, soreness and weakness of waist and knees, insomnia	0.31	3.30		GLG0023
*Lonicera japonica* Thunb	金银花	Caprifoliaceae	Herbal tea	Flower; dry, soaking in water	Flower; decoction	Cold, fever	0.15	3.38		GLG0024
*Stellaria yunnanensis* Franch	千针万线草	Caryophyllaceae	Herbal tea	Whole plant; dry, soaking in water	Whole plant; decoction	Tonifying qi and invigorating spleen, nourishing liver and activating blood circulation	0.15	7.43		GLG0025
*Garcinia esculenta* Y. H. Li	山木瓜	Clusiaceae	Fruits	Fruit; eaten freshly, soaking in wine	Fruit; decoction, soaking in wine	Clearing heat, detoxification, periodontitis, rheumatism	0.31	12.15		DXB0158
*Disporum bodinieri* (Lévl. et Vant.) Wang et Tang	短蕊万寿竹	Colchicaceae	Medicinal diets	Rhizome; cook soup	Rhizome; boiled with meat	Reinforcing qi and tonifying kidney, moistening lung and relieving cough	0.08	0.41		GLG0026
*Disporum uniflorum* Baker ex S. Moore	少花万寿竹	Colchicaceae	Medicinal diets	Rhizome; cook soup	Rhizome; boiled with meat	Reinforcing qi and tonifying kidney, moistening lung and relieving cough	0.08	0.41		
*Ipomoea batatas* (L.) Lamarck	番薯	Convolvulaceae	Vegetables	Tender stem, tender leaf; boiled or stir-fried	Stem; decoction	Constipation	0.23	38.88	DD	
*Cornus capitata* Wallich	头状四照花	Cornaceae	Fruits	Fruit; eaten freshly	Bark; decoction	Hepatitis	0.23	2.43	LC	GLG0028
*Hellenia speciosa* (J.Koenig) S.R.Dutta	闭鞘姜	Costaceae	Medicinal diets	Tender stem; stir-fried	Rhizome; boiled with meat	Anti-inflammatory diuresis, blood stasis detumescence	0.15	1.32	LC	
*Sechium edule* (Jacq.) Swartz	佛手瓜	Cucurbitaceae	Vegetables	Fruit, tender stem, tender leaf, seed; boiled, stir-fried, cook soup	Fruit; decoction	Clearing away heat andtoxic materials,Spleen appetizer, anorexia	0.77	864.00		GLG0029
*Cucurbita moschata* (Duch. ex Lam.) Duch. ex Poiret	南瓜	Cucurbitaceae	Vegetables	Flower, tender stem, tender leaf, fruit; boiled or stir-fried,fried with eggs	Fruit; decoction	Clearing heat and detoxifying, indigestion	0.62	280.80		
*Momordica charantia* L	苦瓜	Cucurbitaceae	Vegetables	Fruit; stir-fried	Fruit; decoction	Clearing away heat andtoxic materials	0.31	21.60		
*Gynostemma pentaphyllum* (Thunb.) Makino	绞股蓝	Cucurbitaceae	Tea substitute	Stem, leaf; dry, soaking in water	Whole plant; decoction	Anti-inflammatory detoxification, cough expectorant	0.23	10.13		GLG0001
*Cucumis hystrix* Chakr	野黄瓜	Cucurbitaceae	Vegetables	Fruit; stir-fried	Stem; decoction	Anti-inflammatory, expectorant, antispasmodic	0.23	6.08		GLG0002
*Momordica cochinchinensis* (Lour.) Spreng	木鳖子	Cucurbitaceae	Vegetables	Fruit; stir-fried	Seed, root, leaf; decoction	Ulcer swelling, dry tinea, bald sores	0.08	0.66		GLG0005
*Cyclanthera pedata* (L.) Schrad	小雀瓜	Cucurbitaceae	Vegetables	Fruit; stir-fried	Fruit; decoction	Clearing heat and detoxifying, cooling blood	0.15	2.03		GLG0018
*Momordica subangulata* Bl	凹萼木鳖	Cucurbitaceae	Vegetables	Fruit; stir-fried	Fruit; decoction	Clearing away heat andtoxic materials	0.08	0.66		GLG0088
*Eleocharis dulcis* (N. L. Burman) Trinius ex Henschel	荸荠	Cyperaceae	Fruits	Bulb; boiled, eaten freshly	Bulb; decoction	Quench thirst, relieve fever	0.23	7.29	LC	GLG0087
*Dioscorea polystachya* Turczaninow	薯蓣	Dioscoreaceae	Staple food substitutes	Tuber; boiled, cook soup	Tuber; decoction	Eliminating phlegm and treating indigestion	0.38	56.70		
*Dioscorea* sp.	薯蓣属某种	Dioscoreaceae	Medicinal diets	Tuber; cook soup	Tuber; boiled with meat	Eliminating phlegm and treating indigestion	0.08	0.50		
*Elaeagnus conferta* Roxb	密花胡颓子	Elaeagnaceae	Fruits	Fruit; eaten freshly	Fruit; decoction	Diarrhea, dysentery, cough	0.08	1.01	LC	
*Elaeagnus umbellata* Thunb	牛奶子	Elaeagnaceae Juss	Fruits	Fruit; eaten freshly	Fruit; decoction	Dispelling wind and dampness, rheumatic joint pain	0.08	0.41	LC	
*Hippophae rhamnoides* subsp. *yunnanensis* Rousi	云南沙棘	Elaeagnaceae Juss	Fruits	Fruit; eaten freshly	Fruit; decoction	stomachache,cough,indigestion	0.08	0.41		
*Elaeocarpus braceanus* Watt ex C. B. Clarke	滇藏杜英	Elaeocarpaceae	Fruits	Fruit; eaten freshly, soaking in wine	Fruit; decoction, soaking in wine	Traumatic injury	0.54	21.26		GLG0089
*Rhododendron decorum* Franch	大白杜鹃	Ericaceae	Vegetables	Flower; boiled or stir-fried	Root; decoction	Clearing damp heat, activating blood and relieving pain	0.15	1.62	LC	
*Sauropus androgynus* (L.) Merr	守宫木	Euphorbiaceae	Vegetables	Tender stem, tender leaf; stir-fried	Tender stem and leaf; decoction	Headache,hypertension	0.08	0.45		
*Senegalia pennata* (L.) Maslin	印度藤儿茶	Fabaceae	Vegetables	Tender leaf; fried with eggs	Tender leaf; decoction	Clearing damp heat	0.38	12.15	LC	GLG0090
*Phaseolus coccineus* L	荷包豆	Fabaceae	Vegetables	Seed; boiled or stir-fried	Seed; decoction	Removing damp-heat	0.62	28.08	LC	
*Pueraria montana* var. *thomsonii* (Bentham) M. R. Almeida	粉葛	Fabaceae	Staple food substitutes	Root; steam, processed into flour	Root; decoction	Fever, headache	0.23	12.15		GLG0059
*Vicia lens* (L.) Coss. et Gern	兵豆	Fabaceae	Staple food substitutes	Stem; dry, processed into flour	Seed; decoction	Constipation	0.31	3.12		
*Tamarindus indica* L	酸角	Fabaceae	Fruits	Fruit; eaten freshly	Fruit; decoction	Curing rheumatism	0.08	0.81	LC	GLG0055
*Pachyrhizus erosus* (L.) Urb	豆薯	Fabaceae	Vegetables	Root; stir-fried, cold and dressed with sauce	Root; decoction	Antialcoholism, lowering blood pressure	0.15	5.85		
*Styphnolobium japonicum* (L.) Schott	槐	Fabaceae	Tea substitute	Seed; soaking in water	Flower, kernel; decoction	Headache, dizziness	0.15	2.70		GLG0088
*Spatholobus suberectus* Dunn	密花豆	Fabaceae	Medicinal diets	Stem; cook soup	Stem; boiled with meat	Expelling pathogenic wind and promoting blood circulation,relaxing tendons and activating collaterals, waist and knee pain	0.31	0.80		
*Caragana sinica* (Buc'hoz) Rehd	锦鸡儿	Fabaceae	Vegetables	Flower; stir-fried, fried with eggs	Bark; decoction	Dispelling wind and activating blood, relieving cough and resolving phlegm	0.08	1.22		
*Guilandina minax* (Hance) G. P. Lewis	喙荚鹰叶刺	Fabaceae	Herbal tea	Seed; soaking in water	Seed; decoction	Tonifying qi	0.08	0.15		GLG0082
*Bauhinia acuminata* L	白花羊蹄甲	Fabaceae	Vegetables	Flower; stir-fried	Flower; decoction	Clearing away heat andtoxic materials,constipation,indigestion	0.08	0.41	LC	
*Castanea mollissima* Blume	板栗	Fagaceae	Nuts	Seed; eaten freshly, roast	Seed; decoction	Stomach vomiting, sore knees	0.23	12.15	LC	
*Gentiana rigescens* Franch. ex Hemsl	滇龙胆草	Gentianaceae	Herbal tea	Whole plant; soaking in water	Whole plant; decoction	Jaundice hepatitis, tonsillitis, laryngopharyngitis	0.38	9.00		GLG0084
*Ginkgo biloba* L	银杏	Ginkgoaceae	Nuts,vegetables	Fruit, inflorescence; cook soup, stir-fried	Fruit; decoction	Children with convulsions, convulsions, skin itching	0.23	27.34	EN	GLG0085
*Gnetum montanum* Markgr	买麻藤	Gnetaceae	Nuts	Seed; roast	Stem and leaf; external application	Falling injury, rheumatic bone pain	0.15	1.35	LC	
*Curculigo orchioides* Gaertn	仙茅	Hypoxidaceae	Tea substitute	Whole plant; soaking in water, cook soup	Rhizome; decoction	Knee cold pain, reinforcing kidney	0.15	13.65		
*Juglans regia* L	胡桃	Juglandaceae	Nuts, oils and fats	Seed; press into oil	kernel; decoction	Asthma, constipation	0.46	32.40	LC	GLG0033
*Ocimum basilicum* var. *pilosum* (Willd.) Benth	疏柔毛罗勒	Lamiaceae	Spices	Stem, leaf; boiled or stir-fried,cold and dressed with sauce	Stem, leaf; decoction	Stomachache,indigestion,enteritis,cold,headache	0.92	630.00		
*Elsholtzia kachinensis* Prain	水香薷	Lamiaceae	Spices,tea substitute	Stem, leaf; boiled or stir-fried,cold and dressed with sauce	Stem, leaf; decoction	Vomiting, diarrhea, colds	0.92	840.00		GLG0044
*Mentha canadensis* Linnaeus	薄荷	Lamiaceae	Spices,tea substitute	Stem, leaf; boiled or stir-fried, cold and dressed with sauce, soaking in water	Stem, leaf; decoction	Wind heat cold, headache	0.77	765.00		DXB0081
*Agastache rugosa* (Fisch. et Mey.) O. Ktze	藿香	Lamiaceae	Vegetables	Tender stem, tender leaf; boiled or stir-fried	Stem, leaf; decoction	Relieving summer-heat,stomachache,headache	0.31	86.40		
*Perilla frutescens* (L.) Britt	紫苏	Lamiaceae	Spices	Stem, leaf; boil, stir-fried	Stem, leaf, seed; decoction	Cough,asthma, constipation	0.15	32.40	LC	DXB0180
*Elsholtzia ciliata* (Thunb.) Hyland	香薷	Lamiaceae	Tea substitute	Stem, leaf; soaking in water	Whole plant; decoction	Cold,headache, stomachache	0.23	24.30		DLZ0160, DXB0055
*Stachys sieboldii* Miquel	甘露子	Lamiaceae	Vegetables	Tuber; boiled or stir-fried	Whole plant; decoction	Pneumonia, wind-heat cold	0.08	2.70		
*Salvia miltiorrhiza* Bunge	丹参	Lamiaceae	Medicinal diets	Rhizome; cook soup	Rhizome; boiled with meat	Activating blood circulation and removing blood stasis, dredging channels and relieving pain	0.08	1.95		
*Holboellia angustifolia* Wallich	五月瓜藤	Lardizabalaceae	Fruits	Fruit; eaten freshly	Fruit; decoction	Falling injury	0.15	0.41		DXB0116
*Decaisnea insignis* (Griffith) J. D. Hooker et Thomson	猫儿屎	Lardizabalaceae	Fruits	Fruit; eaten freshly	Fruit; decoction	Clearing away heat andtoxic materials, hernia	0.08	0.15		DXB0150
*Litsea cubeba* (Lour.) Pers	山鸡椒	Lauraceae	Spices	Fruit; stir-fried	Fruit; decoction	Balsam	0.31	10.80	LC	DLZ0037
*Litsea pungens* Hemsl	木姜子	Lauraceae	Spices	Fruit; stir-fried	Fruit; decoction	Invigorating spleen to promote digestion	0.08	2.70	LC	DLZ0042
*Cinnamomum cassia* Presl	肉桂	Lauraceae	Spices	Bark; cook soup	Bark; decoction	Balsam	0.08	0.26		
*Lindera communis* Hemsl	香叶树	Lauraceae	Oils and fats	Fruit; press into oil	Bark; decoction	Falling injury	0.15	1.62	LC	
*Lilium davidii* Duchartre ex Elwes	川百合	Liliaceae	Vegetables	Bulb; boiled or stir-fried, fried with eggs	Bulb; decoction	Moistening lung to arrest cough	0.31	16.20		
*Cardiocrinum giganteum* (Wall.) Makino	大百合	Liliaceae	Staple food substitutes	Bulb; boiled or stir-fried, fried with eggs	Bulb; decoction	Moistening lung to arrest cough	0.15	8.10		DLZ0029
*Fritillaria cirrhosa* D. Don	川贝母	Liliaceae	Vegetables	Bulb; boiled or stir-fried	Bulb; decoction	Moistening lung to arrest cough	0.38	6.75	VU	
*Lycopodium japonicum* Thunb. ex Murray	石松	Lycopodiaceae	Herbal tea	Whole plant; soaking in water	Whole plant; decoction	Arthralgia	0.15	5.85		
*Hibiscus sabdariffa* L	玫瑰茄	Malvaceae	Tea substitute	Flower; soaking in water	Flower; decoction	Cough, lower blood pressure	0.08	0.84		
*Paris polyphylla* var. *yunnanensis* (Franch.) Hand.-Mzt	滇重楼	Melanthiaceae	Medicinal diets	Tuber; cook soup	Stem; boiled with meat	Throat swelling and pain, snake bites, falling injury	0.31	1.65		
*Toona sinensis* (A. Juss.) Roem	香椿	Meliaceae	Vegetables	Tender stem, tender leaf; stir-fried, fried with eggs	Stem, leaf; decoction	Hemostasis	0.46	58.32	LC	DXB0043
*Artocarpus heterophyllus* Lam	波罗蜜	Moraceae	Fruits	Fruit, seed; eaten freshly, roast	Fruit; decoction	Stimulation of lactation	0.23	15.19		
*Ficus auriculata* Lour	大果榕	Moraceae	Fruits	Fruit; eaten freshly	Fruit; decoction	Stimulation of lactation	0.31	2.70	LC	
*Morus alba* L	桑	Moraceae	Fruits	Fruit; eaten freshly	Fruit; decoction	Cold, cough	0.08	1.01	LC	
*Musa basjoo* Sieb. et Zucc	芭蕉	Musaceae	Vegetables	Tender pseudostem, flower; boiled or stir-fried	Pseudoste; decoction	Cold, stomachache	0.54	88.20	LC	
*Musella lasiocarpa* (Franchet) C. Y. Wu ex H. W. Li	地涌金莲	Musaceae	Vegetables	Tender pseudostem, flower; stir-fried	Flower; decoction	Hemostasis	0.08	0.56	EN	
*Psidium guajava* L	番石榴	Myrtaceae	Fruits	Fruit; eaten freshly	Leaf; decoction	Stop dysentery, stop bleeding, invigorating stomach	0.08	0.29	LC	
*Nelumbo nucifera* Gaertn	莲	Nelumbonaceae	Vegetables	Tender leaf; stir-fried	Leaf; decoction	Palpitation, insomnia	0.08	2.03		
*Bletilla formosana* (Hayata) Schltr	小白及	Orchidaceae	Medicinal diets	Bulb; cook soup	Bulb; boiled with meat	Traumatic bleeding, sores, swelling, skin chapped	0.31	2.25		
*Dendrobium longicornu* Lindl	长距石斛	Orchidaceae	Herbal tea	Succulent stem; soaking in water	Stem; decoction	Eating less, retching, fever does not retreat after illness	0.23	1.27		
*Gastrodia elata* Bl	天麻	Orchidaceae	Medicinal diets	Rhizome; cook soup	Rhizome; boiled with meat	Child convulsion,headache,dizziness	0.23	1.69	VU	
*Passiflora edulis* Sims	鸡蛋果	Passifloraceae	Fruits	Fruit; eaten freshly	Fruit; decoction	Clearing away heat andtoxic materials	0.46	12.15		
*Phyllanthus emblica* L	余甘子	Phyllanthaceae	Fruits	Fruit; eaten freshly, soaking in wine	Fruit; decoction, soaking in wine	Indigestion, abdominal distension,cough, laryngalgia	0.62	121.50	LC	
*Phytolacca acinosa* Roxb	商陆	Phytolaccaceae	Vegetables	Tender stem, tender leaf; stir-fried	Root; decoction	Purge, carbuncle swelling sore virus	0.08	0.78		
*Piper flaviflorum* C. DC	黄花胡椒	Piperaceae	Medicinal diets	Fruit; dry	Fruit; boiled with meat	Toothache	0.54	15.36		
*Plantago asiatica* L	车前	Plantaginaceae	Vegetables	Whole plant; boiled or stir-fried	Whole plant; decoction	clearing away heat andtoxic materials	0.08	18.00		GLG0077
*Cymbopogon citratus* (D. C.) Stapf	香茅草	Poaceae	Herbal tea	Stem, leaf; soaking in water	Whole plant; decoction	Driving wind and dredging collaterals	0.08	4.50		GLG0076
*Zizania latifolia* (Griseb.) Stapf	菰	Poaceae	Vegetables	Tender stem; stir-fried	Stem; decoction	Health care	0.54	4.73		
*Persicaria viscosa* (Buch.-Ham. ex D. Don) H. Gross ex Nakai	香蓼	Polygonaceae	Spices	Whole plant; boiled or stir-fried	Whole plant; decoction	Stomachache, indigestion, infantile malnutrition, rheumatism pain	0.38	216.00		GLG0066
*Fagopyrum dibotrys* (D. Don) Hara	金荞麦	Polygonaceae	Vegetables	Tender stem, tender leaf; boiled or stir-fried	Root; decoction	Clearing away heat andtoxic materials,expelling pus and removing blood stasis	0.23	37.44		DLZ0089
*Fagopyrum tataricum* (L.) Gaertn	苦荞麦	Polygonaceae	Herbal tea	Seed; soaking in water	Seed; decoction	Moisturizing bowel, constipation	0.31	6.24		
*Pleuropterus multiflorus* (Thunb.) Nakai	何首乌	Polygonaceae	Medicinal diets	Root; cook soup	Root; boiled with meat	Moisturizing bowel, constipation	0.08	0.41		
*Portulaca oleracea* L	马齿苋	Portulacaceae	Vegetables	Whole plant; boiled or stir-fried	Whole plant; decoction	Snake bite, hematochezia	0.08	9.36	LC	
*Coptis teeta* Wall	云南黄连	Ranunculaceae	Medicinal diets	Root; cook soup	Root; boiled with meat	Stomachache	0.23	0.83	EN	
*Thalictrum alpinum* L	高山唐松草	Ranunculaceae	Medicinal diets	Root; cook soup	Root; boiled with meat	Infantile malnutrition, infantile convulsions	0.31	1.60		
*Aconitum carmichaelii* Debeaux	乌头	Ranunculaceae	Medicinal diets	Root; cook soup	Tuber; boiled with meat	Wind-cold syndrome, joint pain	0.08	0.30		
*Hovenia acerba* Lindl	枳椇	Rhamnaceae	Fruits	Fruit; eaten freshly	Fruit; decoction	Rheumatism, hangover	0.31	6.75	LC	DXB0100
*Pseudocydonia sinensis* (Thouin) C. K. Schneid	木瓜	Rosaceae	Fruits	Fruit; cold and dressed with sauce, dry	Fruit; decoction	Rheumatism, joint pain	0.85	386.10	LC	DLZ0124
*Prunus salicina* Lindl	李	Rosaceae	Fruits	Fruit; eaten freshly	Fruit; decoction	Cough, diarrhea	0.08	4.05	LC	
*Rosa rugosa* Thunb	玫瑰	Rosaceae	Tea substitute	Flower; soaking in water	Flower; decoction	Wind-heat cold, laryngalgia	0.08	1.01		
*Prunus mume* Siebold & Zucc	梅	Rosaceae	Fruits	Fruit; eaten freshly	Fruit; decoction	Cough, diarrhea	0.08	4.05	LC	
*Malus asiatica* Nakai	花红	Rosaceae	Fruits	Fruit; eaten freshly	Fruit; decoction	Stopping thirst and generating fluid, digesting food and resolving stagnation	0.15	0.34	DD	
*Rubus sumatranus* Miq	红腺悬钩子	Rosaceae	Fruits	Fruit; eaten freshly	Root; decoction	Clearing away heat andtoxic materials, diuresis	0.08	0.34		DXB0107
*Dimetia scandens* (Roxb.) R. J. Wang	藤耳草	Rubiaceae	Herbal tea	tender stem, tender leaf; cook soup	Stem and leaf; decoction	Lung disease, pneumonia	0.15	6.60		
*Scleromitrion diffusum* (Willd.) R. J. Wang	白花蛇舌草	Rubiaceae	Herbal tea	Stem, leaf; soaking in water	Stem and leaf; decoction	Anti-inflammatory, laryngalgia	0.15	4.95		
*Gardenia jasminoides* Ellis	栀子	Rubiaceae	Herbal tea	Fruit; soaking in water	Fruit; decoction	Promote digestion	0.15	3.71		
*Citrus medica* L	香橼	Rutaceae	Tea substitute	Fruit; dry, soaking in water	Fruit; decoction	Excessive phlegm,cough	0.54	56.70	LC	
*Zanthoxylum armatum* DC	竹叶花椒	Rutaceae	Spices	Fruit; dry	Fruit; decoction	Rheumatoid arthritis, toothache, bruising pain	0.31	32.40	LC	DLZ0143
*Houttuynia cordata* Thunb	蕺菜	Saururaceae	Spices	Whole plant; boiled or stir-fried, cold and dressed with sauce	Whole plant; decoction	Enteritis, dysentery	1.00	1365.00		DLZ0023
*Schisandra neglecta* A. C. Smith	滇藏五味子	Schisandraceae	Fruits	Fruit; eaten freshly, soaking in wine	Fruit; soaking in wine	Palpitation, insomnia	0.15	2.93		DLZ0039
*Buddleja officinalis* Maxim	密蒙花	Scrophulariaceae	Dyes	Flower; dye	Flower; decoction	Clearing away heat andtoxic materials,improving acuity of vision	0.08	0.29	LC	
*Smilax china* L	菝葜	Smilacaceae	Vegetables	Tender stem, tender leaf; stir-fried	Stem; decoction	Rheumatalgia	0.08	1.04		
*Solanum americanum* Miller	少花龙葵	Solanaceae	Vegetables	Leaf; boiled or stir-fried	Leaf; decoction	Laryngalgia	0.31	54.00		
*Cyphomandra betacea* Sendt	树番茄	Solanaceae	Vegetables	Fruit; stir-fried	Fruit; decoction	Invigorate the spleen and stomach	0.69	31.59		GLG0011
*Solanum torvum* Swartz	水茄	Solanaceae	Vegetables	Fruit; stir-fried	Fruit, leaf; decoction	Improving acuity of vision, sore toxins	0.23	7.02		GLG0017
*Lycium chinense* Miller	枸杞	Solanaceae	Vegetables	Tender leaf; boiled or stir-fried	Fruit; decoction	Lumbar and knee pain, dizziness, tinnitus	0.23	7.80		
*Solanum aethiopicum* Linnaeus	红茄	Solanaceae	Vegetables	Fruit; stir-fried	Fruit; decoction	Inflammatory pain, stomach pain, lymph nodes	0.77	23.40		GLG0019
*Camellia reticulata* Lindl	滇山茶	Theaceae	Oils and fats	Seed; fried with eggs	Seed; decoction	Clearing away heat andtoxic materials, prevention of cardiovascular disease	0.08	0.33	DD	
*Zingiber officinale* Roscoe	姜	Zingiberaceae	Spices	Rhizome; boiled or stir-fried	Rhizome; decoction	Stomachache, vomiting, diarrhea, colds	0.46	64.80	DD	
*Amomum tsaoko* Crevost et Lemarie	草果	Zingiberaceae	Spices	Fruit; boiled	Fruit; decoction	Stomachache, fever	0.54	18.90		GLG0020
*Amomum villosum* Lour	砂仁	Zingiberaceae	Spices	Fruit; boiled with meat	Fruit; decoction	Stomachache	0.15	5.40	LC	
*Hedychium coronarium* Koen	姜花	Zingiberaceae	Vegetables	Flower; fried with eggs	Rhizome; decoction	Headache,body pain, rheumatic pain, falling injury	0.08	0.90	DD	
**Gymnosperm**										
*Pinus armandii* Franch	华山松	Pinaceae	Nuts	Seed; stir-fried	Seed; decoction	Pulmonary dryness, cough, constipation	0.23	4.68	LC	
**Fern**										
*Diplazium esculentum* (Retz.) Sm	菜蕨	Athyriaceae	Vegetables	Tender stem, tender leaf; boiled or stir-fried	Stem, leaf; decoction	Clearing away heat andtoxic materials	0.23	2.17	LC	DLZ0027
*Osmunda japonica* Thunb	紫萁	Osmundaceae	Vegetables	Tender stem, tender leaf; boiled or stir-fried	Tender stem and leaf; decoction	Cold, carbuncle swelling virus	0.46	77.76		DXB0106
*Sceptridium ternatum* (Thunb.) Y. X. Lin	阴地蕨	Ophioglossaceae	Herbal tea	Whole plant; soaking in water, cook soup	Whole plant; decoction	Trachitis, pneumonia	0.23	16.20		
*Angiopteris esculenta* Ching	食用观音座莲	Marattiaceae	Staple food substitutes	Rhizome; processed into flour	Rhizome; external application	Pruritus	0.08	0.29		DXB0103
*Diplazium viridissimum* Christ	深绿短肠蕨	Athyriaceae	Vegetables	Tender stem, tender leaf; boiled or stir-fried	Tender stem and leaf; decoction	Clearing away heat andtoxic materials	0.15	12.96		
*Pteridium aquilinum* var. *latiusculum* (Desv.)Underw.ex Heller	蕨	Dennstaedtiaceae	Vegetables	Tender leaf; stir-fried, cold and dressed with sauce	Whole plant; decoction	Clearing away heat andtoxic materials,diuresis	0.77	90.00		
*Rhodobryum giganteum* Par	暖地大叶藓	Bryaceae	Medicinal diets	Whole plant; cook soup	Whole plant; boiled with meat	Heart disease such as palpitations, palpitations	0.31	2.40		
*Lygodium japonicum* (Thunb.) Sw	海金沙	Lygodiaceae	Herbal tea	Whole plant; soaking in water	Stem and leaf; decoction	Balsam	0.15	3.60		
**Lichen**										
*Thamnolia vermicularis* (Sw.) Ach. ex Schaer	雪茶	Thamnoliaeeae	Tea substitute	Thallus; soaking in water	Thallus; decoction	Cough, fever	0.23	5.06		
*Ramalina fastigiata* (Pers.) Ach	从生树花	Ramalinaceae	Vegetables	Thallus; cold and dressed with sauce, stir-fried	Thallus; decoction	Clearing away heat andtoxic materials	0.69	43.88		

**Fig. 2:**
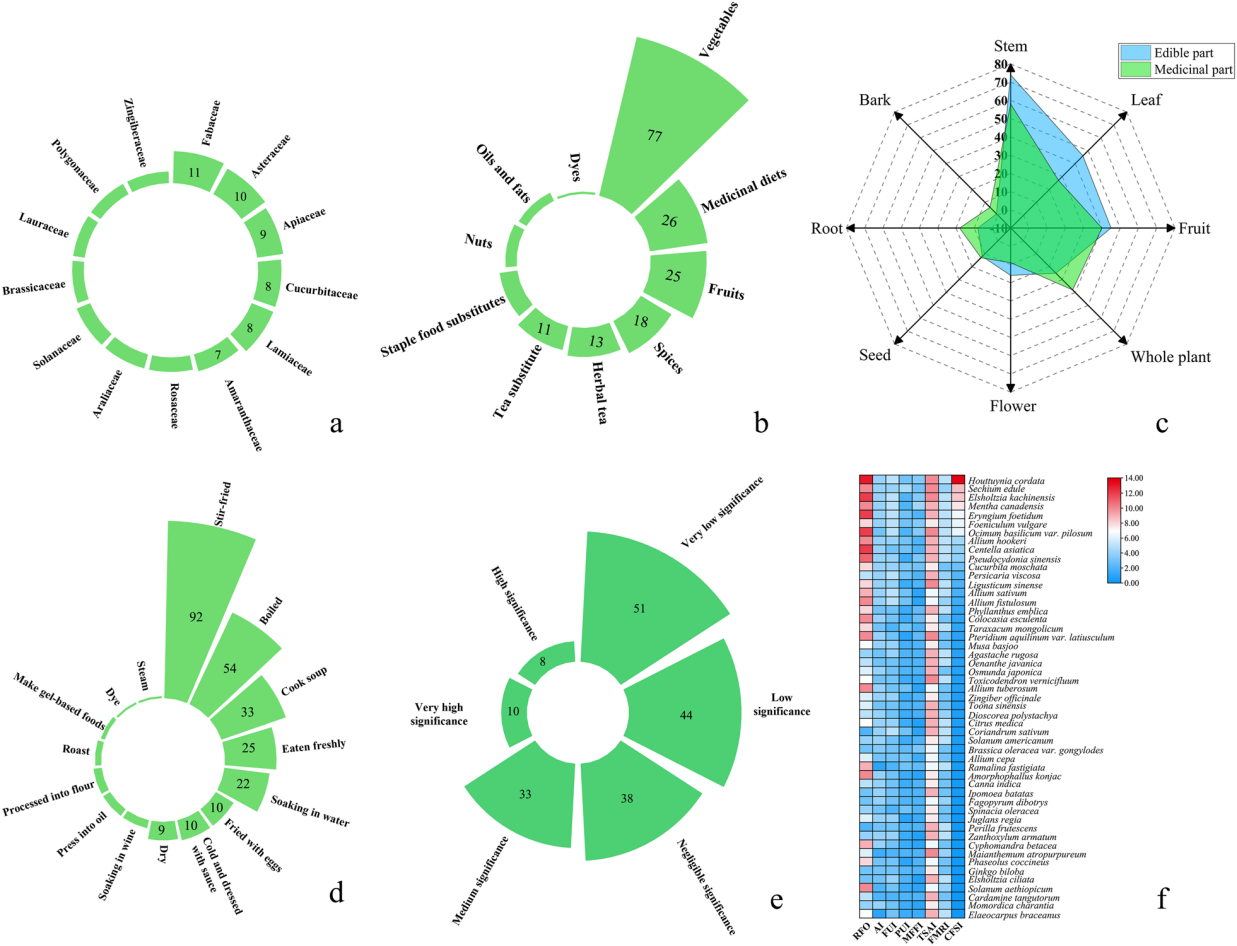
Diversity of medicinal food plants. **a** Distribution of medicinal food plants’ family; **b** distribution of food types in market survey results; **c** the edible and medicinal parts of medicinal dietary plant; **d** the mode of consumption of medicinal food plants; **e** the CFSI of medicinal food plants; **f** the top 50 CFSI of medicinal food plants

A total of 184 medicinal food plant species belonging to different 83 families were recorded. The plant species recorded in the study area were presented in Table 2, arranged in alphabetical order for families and entities. The most frequently used families Fabaceae, Asteraceae and Apiaceae were the most genera, with 11, 10 and 9 species, respectively. Followed by Labiatae and Cucurbitaceae, both containing 8 species. There are seven species in the Amaryllidaceae family. The remaining families were represented by 6 or fewer entities (Fig. 2a). In previous studies, these families were also reported to be widely used by ethnic minorities in northwest Yunnan, China [60].

Different medicinal and edible parts are used by the residents in Gaoligong Mountains area, such as the seeds, leaves, stems, flowers, seeds, and other eight parts. In terms of edible parts, the most commonly used parts are stems, leaves, fruits, and flowers. These parts of the plant are used more as edible parts than medicinal parts. In terms of medicinal parts, the most commonly used parts are whole plants, bark, roots, and seeds. Plants that utilize these parts for medicine more than for edible purposes (Fig. 2c).

This shows that people are more inclined to reuse for edible plants, and most of the used parts are sustainable. For medicinal plants, local people use more unsustainable parts. Some previous studies have also found a similar phenomenon, which may be related to the accumulation of active ingredients in roots, bark, and other parts [60].

### Classification of medicinal food plants

According to the purpose of the medicinal food plants investigated and combined with the food-preparing methods of the local people, the edible types of all medicinal food plants can be divided into ten categories: vegetables, medicinal diet, fruits, spices, herbal tea, tea substitute, staple food substitute, nuts, oils and dyes.

Among the 184 species of MFPs in the Gaoligong Mountains area, the most used food categories by local people are vegetables, with 77 species, followed by medicinal diet and fruits, with 26 and 25 species respectively, followed by spices, herbal tea, tea substitute, substitute for staple food, nuts, and edible oils. Dye use is the least category, with only 1 species (Fig. 2b).

### Vegetables

**Fig. 3:**
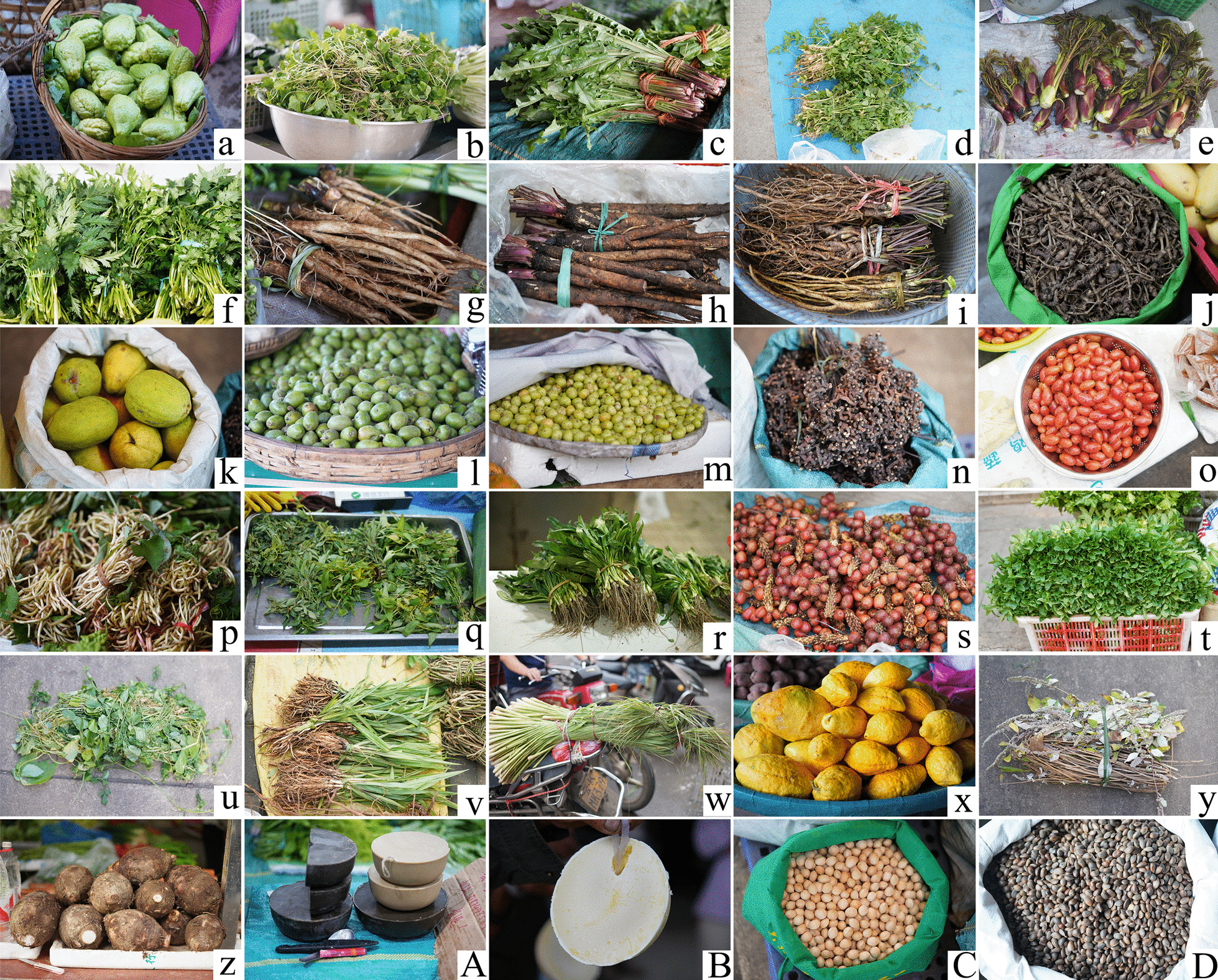
Plants with high Cultural food significance index (CFSI) index in Gaoligong Mountains area. **a** *Sechium edule*, **b** *Centella asiatica*, **c** *Taraxacum mongolicum*, **d** *Oenanthe javanica*, **e** *Aralia chinensis*, **f** *Ligusticum sinense* ‘Chuanxiong’, **g** *Cirsium spicatum*, **h** *Pimpinella candolleana*, **i** *Arctium lappa*, **j** *Panax japonicus*, **k** *Pseudocydonia sinensis*, **l** *Elaeocarpus braceanus*, **m** *Phyllanthus emblica*, **n** *Hovenia acerba*, **o** *Elaeagnus conferta*, **p** *Houttuynia cordata*, **q** *Persicaria viscosa*, **r** *Eryngium foetidum*, **s** *Amomum tsaoko*, **t** *Mentha canadensis*, **u** *Gynostemma pentaphyllum*, **v** *Curculigo orchioides*, **w** *Cymbopogon citratus*, **x** *Citrus medica*, **y** *Elsholtzia rugulosa*, **z** *Colocasia esculenta*, **A** *Toxicodendron vernicifluum*, **B** *Lindera communis*, **C** *Ginkgo biloba*, **D** *Pinus armandii*

Vegetables are the largest type of medicinal food plants used by residents in the Galigong Mountains area, with a total of 77 kinds. The main edible parts of vegetables are stems and leaves, and the cooking methods mainly include stir-fried, boiled, cooked soup, fried with eggs and so on (Fig. 2d). Examples include *Sechium edule*, *Centella asiatica*, *Taraxacum mongolicum*, *Oenanthe javanica*, and *Aralia chinensis* (Fig. 3a–e). Vegetables can supplement dietary fiber, vitamins, amino acids and a variety of mineral elements for the human body, so they are an indispensable food every day [61]. The great market demand and high healthcare value make the Gaoligong Mountains area have unlimited development potential for the MFPs as vegetables, which can increase the income of local residents and improve their living conditions.

### Medicinal diet

Medicinal diet is the second category of MFPs used by local people in Gaoligong Mountains area, with a total of 26 species. The main part of medicinal food used is stem, and the main cooking method is boiling with meat, such as *Ligusticum sinense* 'Chuanxiong', *Cirsium spicatum*, *Pimpinella candolleana*, *Arctium lappa*, and *Panax japonicus* (Fig. 3f–j). The social and economic level of Gaoligong Mountain area is not high, and fighting against the harsh natural environment is a compulsory course for local residents, which leads to their high demand for medicinal diet and accumulation of a large amount of traditional knowledge related to MFPs with national characteristics, which is of great promotion value in contemporary times [57].

### Fruit

Fruits are the third category of MFPs used by Gaoligong Mountains residents, with a total of 25 species. Most of the plants used as fruits are not processed by local residents and are eaten freshly, such as *Pseudocydonia sinensis*, *Elaeocarpus braceanus*, *Phyllanthus emblica*, *Hovenia acerba*, and *Elaeagnus conferta* (Fig. 3k–o). The unique natural conditions and different climate types in Gaoligong Mountains region making it suitable for the growth of different fruits. Many fruits rich in nutrition and medicinal value are very popular in the local area.

### Spices

Spices are the fourth category of MFPs used by Gaoligong Mountain residents, with a total of 18 species. Spices’ main used parts are stems, leaves and fruits, the processing methods are stir-fried and boiled. Such as *Houttuynia cordata*, *Persicaria viscosa*, *Eryngium foetidum*, *Amomum tsaoko*, and *Mentha canadensis* (Fig. 3p–t). Some spices, are popular in the kitchen of local residents, can not only add flavor to the dishes on the table, but also have an antiseptic and medicinal effect.

### Herbal tea and tea substitute

Herbal tea and substitute tea are the fifth and sixth category of MFPs in Gaoligong Mountains area, with 13 kinds and 11 kinds respectively. Their main use parts are stems and leaves, the main processing method is soaking in water. Such as *Gynostemma pentaphyllum*, *Curculigo orchioides*, *Cymbopogon citratus*, *Citrus medica*, and *Elsholtzia rugulosa* (Fig. 3u–y). Many studies have proved that herbal tea and substitute tea is rich in abundance active substances, which are good for human body. These teas also have medicinal properties, which can help fight disease and condition the body [49].

### Substitutes for staple food, nuts, oil and fats, and dyes

Substitutes for staple food, nuts, oils and fats, and dyes are the seventh, eighth, ninth, and tenth categories of MFPs, with 8, 5, 4, and 1 species, respectively. The main parts of staple food substitute are stems (including rhizomes or tubers) and roots, and the main processing way is boiling and processing into starch or gel food, such as *Colocasia esculenta* (Fig. 3z). Many species are substitutes for food in Gaoligong Mountains area, reflecting the diversity and demand of staple food of local residents, which can help local residents to survive the period of food shortage to a certain extent [44, 62]. *Buddleja officinalis* is the only plant used as dye, and its flowers (inflorescences) are consumed (Fig. 3A–D).

### Quantitative evaluation of medicinal food plants

The CFSI were different among different MFPs. The minimum value was 0.15 while the maximum value was 1365. According to Pieroni’s analysis of the index value, wild edible plants can be divided into six categories according to the sizes of CFSI value, which are: very high (CFSI = 300 and above), high (CFSI = 100–299), medium (CFSI = 20–99), low (CFSI = 5–19), very low (CFSI = 1–4), negligible (CFSI < 1) [59]. The numbers of plants in these six groups were different. Most belonged to the following three groups of very low, low, negligible, and Medium, with 51 species, 44 species, 38 species, and 33 species respectively. This was followed by the very high and high groups, with 10 and 8 species, respectively (Fig. 2e).

The CFSI value was ranked, and the top 51 MFPs (with very high significance (10 species), high significance (8 species), and medium significance (33 species)) were evaluated of which heat maps were made (Fig. 2f). The comparison of CFSI and RFO showed that 38 out of 51 plants were the same, indicating that the plants evaluated and screened by these two indexes were very consistent and had relatively high reliability. The grades of some species were different according to different indexes, indicating that different indicators have different importance attribute to the evaluation. For example, CFSI also included the evaluation of taste and function.

## Discussion

Due to the advantaged natural environment and rich plant resources, Gaoligong Mountains area is known as one of the world’s 25 biodiversity hotspots [41]. The altitude difference of the Gaoligong Mountains area is as high as 4038 m, forming significant climatic changes in the vertical direction [63, 64]. From the top of the mountain to the valley, there are four vertical climatic zones, such as cold zone, subcold zone, temperate zone, and subtropical zone, which have obvious vertical characteristics in terms of temperature, precipitation, soil nutrients and light intensity, thus creating a rich variety of medicinal food plants [65].

The Gaoligong Mountains area is also a multi-linguistic settlement area. There are 16 linguisitc groups in the region, including Han, Dai, Lisu, Pumi, Jingpo, Nu, Dulong, Achang, Naxi, Hui, Bai, Miao, Wa, Yi, Tibetan, and De’ang [66–68]. According to the records, all the linguistic groups living in the Gaoligong Mountains area have the tradition use of MFPs. Local people incorporate their customs, religious beliefs, and dietary ways into the plants for MFPs. Many MFPs used in Gaoligongshan areas have been reported to have high nutritional value. Previous studies have proved that the use of MFPs by ethnic minorities in the Nujiang area is scientific in terms of food nutrition, chemical composition, and pharmacological activity, such as *Caryota obtusa*, *Maianthemum atropurpureum*, *Toxicodendron vernicifluum*, and *Angiopteris esculenta* [13, 17, 18, 27]. Some MFPs are very important in the local area. For example, *Houttuynia houttuynia* is rich in protein, fat, polysaccharide, vitamin and other components, which has high development potential [69, 70]. The local people grow *Sechium edule* in their home gardens and eat their roots, tender stems, leaves, seeds, and fruits. This vegetable has a long consumption cycle and can be used not only as a vegetable, but also for medicinal purposes to clear heat and toxic materials away, and also has a good effect on anorexia. The different cultures and living environments of different linguistic groups lead to different demands for MFPs, which results in the differences of MFPs in different regions [71, 72].

### The potential value of traditional knowledge associated with medicinal food plants

**Fig. 4:**
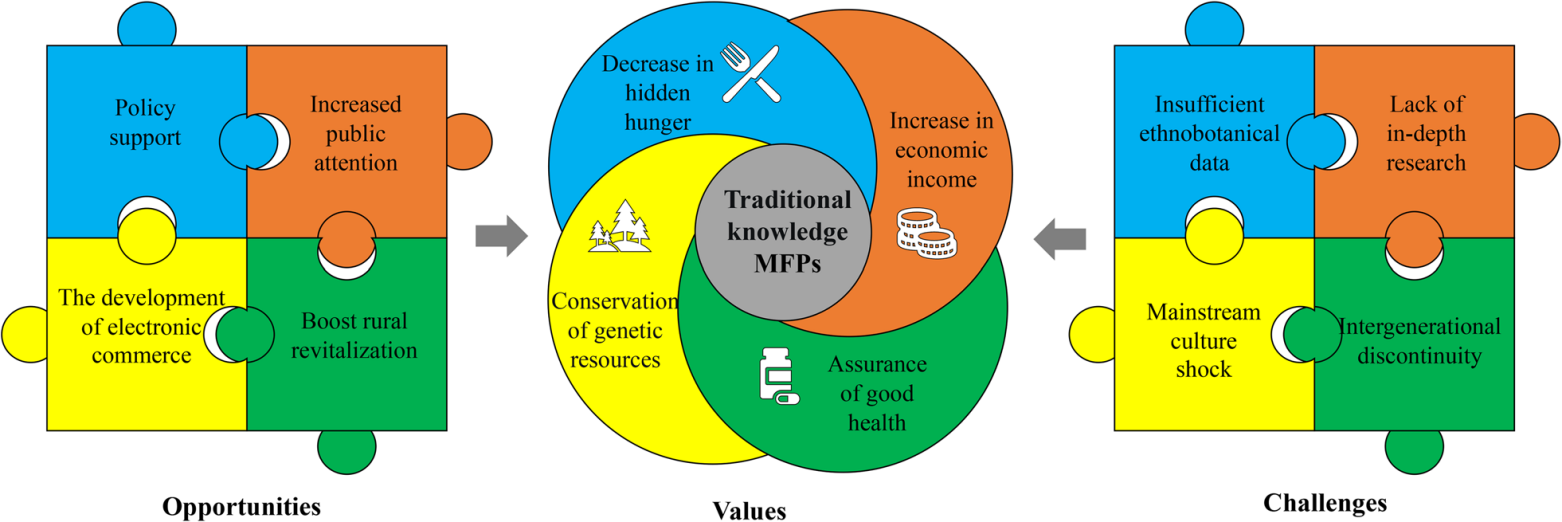
The potential value, opportunities, and challenges for traditional knowledge associated with medicinal food plants

Limited transportation conditions and complex geographical environment in Gaoligong Mountains area make local residents more dependent on local natural resources, and these abundant MFPs resources play an indispensable role in their daily life (Fig. 4).

Firstly, traditional knowledge associated with MFPs can alleviate hidden hunger in remote areas. It can not only provide local residents with essential nutrients, but also ensures their food security [2, 53].

Secondly, traditional knowledge associated with MFPs plays an important role in safeguarding the health and disease resistance for residents in remote areas. In the process of getting along with the local natural environment for thousands of years, the local peoples have accumulated a large amount of traditional knowledge related to disease prevention and treatment and formed the methods of using MFPs with local cultural characteristics [73–75]. These methods can still reflect the survival wisdom of the working people and its unique and indispensable value in modern times. With the growing desire of modern people to pursue a healthy life, people's need for food is no longer just to satisfy their hunger. Local abundant MFPs resources have medicinal value and healthcare function, for example, taking *Ligusticum sinense* 'Chuanxiong' can promote blood circulation, and taking *Pimpinella candolleana* internally can treat jaundice hepatitis and acute cholecystitis [39, 76], which just meet people's needs in this respect [46, 67].

Thirdly, MFPs resources can provide local residents with a source of livelihood and solve their livelihood problems. And these economic sources can ensure their basic living needs, but also maintain the local national culture and traditional lifestyle of the important material basis. Therefore, MFPs have promoted the development of local economy and brought fruitful results for poverty alleviation and rural revitalization [77, 78].

Finally, These MFPs resources have unique and excellent characteristics in all aspects, such as drought tolerance, heat tolerance, and cold tolerance. If the use of its own genetic characteristics, as genetic material, to create a better variety, artificial cultivation, play its greater value. If the genetic characteristics of MFPs are more fully utilized as genetic material, better varieties can be created and artificially cultivated, which can exert greater value [79, 80].

### Opportunities and challenges for the protection of medicinal food plants

MFPs face multiple threats and opportunities in the Gaoligong Mountains area. Nowadays, environmental pollution and changes in diet structure make cardiovascular, cerebrovascular, immune and other diseases frequent and younger, and people’s requirements for a healthy life are constantly increasing. Dietary therapy instead of drug therapy can not only avoid the harmful residue of drugs in the human body, but also reduce people's economic burden [81, 82]. As human beings pay more and more attention to health care, it will become a trend to use MFPs to protect their health, which is a good news for MFPs; another reason is the support by national policies, which have introduced a series of regulations to promote the development of the dual-use industry of MFPs [83] (Fig. 4).

The rapid development of e-commerce and the role of MFPs in promoting rural revitalization are also important factors. With the rise of short video platforms, live-streaming provides a new development opportunity, builds a platform for the sales of MFPs and their products, saves operating costs, and injects new vitality into the Gaoligong Mountains market [84, 85].

Although the widely use brings opportunities, at the same time, medicinal food plants are also facing challenges and threats. (1) Lacking of traditional knowledge database associated with MFPs. At present, there is insufficient ethnobotanical data on MFPs, and relevant traditional knowledge is rapidly disappearing. We should conduct a more comprehensive investigation, recording and cataloging of the endangered knowledge through ethnobotanical methods. (2) Lacking of in-depth research on traditional knowledge associated with MFPs. We should use modern ethnobotanical methods to explain the folk use of MFPs with scientific evidence revealing the scientific basis, which is conducive to traditional knowledge protection and sustainable development. (3) With the acceleration of urbanization, the inheritance of traditional knowledge is also seriously threatened. The traditional knowledge left by the older generation has no one to inherit it. Many studies in different places have emphasized this problem [65, 86, 87]. The foreign culture shock is also an important reason for the loss of traditional knowledge, which means that the cultural dissemination of Han and other ethnic groups has led to local people gradually ignoring and forgetting their own traditional knowledge, just like the loss situation of traditional knowledge related to medicinal plants in other regions [88].

Through the statistics of the endangered situation of MFPs in Gaoligongshan area, it was found that there were one endangered species, *Coptis teeta*, and two vulnerable species, *Gastrodia elata* and *Fritillaria cirrhosa*. These medicinal plants are in urgent need of protection. Some taboo cultures of the ethnic people in Gaoligongshan area are of great significance to the protection and sustainable utilization of both MFPs. On the one hand, it is reflected in the customary law or religious taboo derived from their historical and cultural situatedness, some ethnic people worship some plants as totems and prohibit cutting them down. For example, the Lisu, Nu and Dulong ethnic groups have provisions that prohibit cutting “sacred trees” [10]. On the other hand, due to the cultural taboos, people will restrict the use of MFPs to achieve sustainable utilization. Dulong people have a very strong cultivation and management culture of *Caryota obtusa*. During their farming activities, the Dulong people remove weeds from *C. obtusa* or transplant some seedlings from the mountains into their home gardens or next to the villages. Generally, for major events like building a house or getting married, *C. obtusa* is often planted in the home gardens in case of famine. It is not only related to the local environmental conditions but also to the traditional culture, which helps maintain the population of *C. obtusa* [43–45]. In addition, when collecting medicinal plants, the ethnic people will follow the principle of “gathering the large and leaving the small” [58].

Traditional knowledge associated with MFPs in the Gaoligong Mountains area are precious wealth passed down from generation to generation by local residents. This traditional knowledge is the guarantee of people's livelihood and health, and it is also the culture, customs, and even the blood of the nation. If it is not investigated, cataloged, organized and studied in time, this knowledge will disappear forever, and the consequences of losing it will be immeasurable.

## Conclusion

This study is the first ethnobotanical survey of MFPs in Gaoligong Mountains areas. A total of 184 MFPs species belonging to 83 families used by local people were investigated and recorded, reflecting local people have rich traditional knowledge about MFPs, which plays an important role in their livelihoods. Some MFPs like *Houttuynia cordata*, *Eryngium foetidum*, *Sechium edule*, *Centella asiatica*, *Pseudocydonia sinensis*, and others had high CFSI. These findings have guiding significance for protection of traditional knowledge associate with MFPs, facilitation of scientific utilization of MFPs to meet local people's needs for a healthy life in the Gaoligong Mountains area.

### Supplementary Information

**Additional file 1**. **Table S1**. CFSI data.

## Data Availability

All data generated or analyzed during this study was included in this published article (along with the supplementary files).
